# Maternal and child healthcare coverage and trends: refugee vs. non-refugee districts in Uganda

**DOI:** 10.1186/s13031-026-00780-7

**Published:** 2026-03-11

**Authors:** Rogers Nsubuga, Rornald Muhumuza Kananura, Ronald Wasswa, Catherine Birabwa, Jimmy Ogwal, Winfred Dotse-Gborgbortsi, George Mwinnyaa, Amanuel Abajobir, Julius Kisozi, Alypio Nyandwi, Ties Boerma, Peter Waiswa, Kristine Nilsen

**Affiliations:** 1 Center of Excellence for Maternal, Newborn and Child Health, Department of Health Policy Planning and Management, Makerere University School of Public Health, Kampala, Uganda; 2 Department of Research, Infectious Diseases Institute, Makerere University, Kampala, Uganda; 3 Africa Population and Health Research Center, Nairobi, Kenya; 4 Ministry of Health, Kampala, Uganda; 5 Department of Social Statistics and Demography and WorldPop, University of Southampton, Southampton, UK; 6 Department of Epidemiology, Bloomberg School of Public Health, Johns Hopkins University, Baltimore, MD US; 7 Instittue of Global Health, University of Manitoba, Winnipeg, MB Canada; 8 United Nations High Commissioner for Refugees, Kampala, Uganda

**Keywords:** Refugee-Hosting districts, Routine data, Coverage Composite Index

## Abstract

**Background:**

Uganda hosts the largest refugee population in Africa, which exerts much pressure on the district health systems. While refugee-hosting districts (RH) receive targeted investments, the extent to which these influence maternal and child health (MCH) service coverage remains unclear. Using routine facility data, we examined differences in MCH coverage and trends between RH and non-refugee-hosting (non-RH) districts and also explored the effects of government health financing and health system performance on MCH coverage.

**Methods:**

We conducted a retrospective analysis utilizing routine health facility MCH data from the Uganda District Health Information System and district-level government Primary Healthcare (PHC) expenditure data from 2020 to 2023. MCH indicators were ANC1st trimester, ANC4, Institutional deliveries, mothers’ Post-natal care (PNC), Measles1 and DPT3 vaccination. We computed a composite coverage index (CCI), health systems performance z-score and compared trends across RH and non-RH districts. Mixed Effects Models assessed the association between government expenditure, RH-status, health system performance over the years.

**Results:**

RH districts consistently had modestly higher coverage of ANC1st trimester, ANC4, Institutional deliveries, PNC, Measles vaccination and CCI trends. Government expenditure was significantly higher in RH districts and refugee-dominant (RD) districts (*p* < 0.001 vs. *p* = 0.007). Refugee-dominant districts had higher but non-significant MCH coverage. Unadjusted models of MCH indicators and CCI were positively influenced by government financing and health systems performance z-score except for DPT3 and Measles, respectively. Adjusted models revealed that ANC4 coverage was 7.4% points higher in RH districts (7.42; 95% CI:0.753, 14.090; *p* = 0.029) and increased by 3.6% points for every unit increase in z-score (3.60; 95% CI: 0.729, 6.462; *p* = 0.014). CCI increased by 1.6% points and 2.3% points for every unit increased in the government expenditure and z-score respectively (1.55; 95% CI: 0.310, 2.788; *p* = 0.014) vs. (2.31; 95% CI: 0.642, 3.975; *p* = 0.007).

**Conclusion:**

Novel approach - leveraging routine facility data, revealed MCH coverage was modestly consistently higher in RH districts over the years and RH status influenced ANC4 coverage. Overall district-CCI depended on Government investment and health systems performance implying increase in PHC financing could be a key driver to universal district-level improvement.

## Background

Globally, displacement due to conflict and crises has reached unprecedented levels. By the end of 2024, an estimated 122 million people worldwide were forcibly displaced, including 43 million refugees [1, 2]. Majority (86%) of refugees originate from and are hosted in low- and middle-income countries (LMICs), particularly in sub-Saharan Africa (SSA) and the Asia Pacific region [3]. Sub-Saharan Africa alone saw refugee numbers rise from 2.2 million in 2010 to 6.3 million in 2019, largely driven by crises in countries such as South Sudan, Somalia, Democratic Republic of Congo (DRC), and Central African Republic [2]. Within this region, Uganda, Kenya, Ethiopia, and Sudan host the highest numbers of refugees.

Despite their generosity, these host countries face considerable challenges due to fragile and under-resourced health systems [4, 5]. Ensuring access to quality health services for both refugees and host populations remains a shared responsibility between governments and humanitarian actors [6, 7], but with a heavy or primary responsibility being on the host-government. Health service delivery in such settings often requires integrated and sustainable approaches that strengthen local health systems to absorb sudden and massive population inflows [8, 9].

Uganda hosts the 6th largest refugee population in the world and largest in Africa, with over 1.8 million refugees and asylum seekers mostly from South Sudan (54%) and DRC (32%) [10]. About 1.47 million refugees and asylum seekers reside in rural-based refugee settlements hosted by 12 districts including Isingiro in the South-Western region, Kamwenge, Kikuube, Kiryandongo, Kyegegwa in the Mid-Western region, while Adjumani, Koboko, Lamwo, Obongi, Yumbe, Madi Okollo and Terego in West Nile region.

Over the past five years, refugee-hosting (RH) districts experienced huge growth in the refugee population mainly due to new arrivals and births. Between 2020 and 2021, about 112,000 individuals were newly registered in refugee settlements, and 260,000 new arrivals between 2022 and 2023 [10–12]. This continuous influx, combined with a natural population growth (3% annually) [13], put significant pressure on the already limited services in RH districts, including health especially in the area of health service utilization. Rapid population expansion often leads to shortages and reduced availability of essential health services in low-resource settings, particularly where systems are already fragile (Appendix: Fig. 5: Conceptual Framework). These effects could be more pronounced in districts where refugees make up a large share of the population such as; Obongi (72%), Adjumani (48%), and Madi Okollo and Terego (35%) as compared to those with lower refugee-population share, such as Kiryandongo (12%) [10].

Maternal and child health (MCH) is a central indicator of overall health system performance, particularly in low-resource and humanitarian settings where pregnancy and early childhood carry heightened vulnerability, and disruptions in access to MCH essential services can lead to preventable morbidity and mortality [14]. More than half of global maternal deaths occur in fragile and crisis-affected contexts, with sub-Saharan Africa bearing 86% of the global burden [15]. In Uganda and similar SSA contexts, MCH indicators including antenatal care (ANC), skilled birth attendance, child immunization, and postnatal care are key proxies for system functioning because they require continuity of care, reliable commodities, and timely service delivery. However, the availability and quality of these essential services can be compromised by the unique pressures within RH districts: increased demand, overstretched facilities, and fluctuating resource allocation. The selected indicators in this study align with global monitoring frameworks, including the Sustainable Development Goals (SDGs), which emphasize universal coverage of reproductive, maternal, newborn, and child health services (WHO SDG 3.1 and 3.8). Assessing these indicators at district level is essential for identifying inequities, understanding the impact of refugee inflows on service utilization, and guiding health system investments.

While Uganda has made commendable efforts to integrate refugees into the national health system, challenges persist. Under the Refugee Act of 2006, refugees are entitled to equitable access to public health services [16]. Since 2017, Uganda adopted the Comprehensive Refugee Response Framework (CRRF) and the Global Compact on Refugees (GCR) to strengthen service delivery and promote shared responsibility [17]. These policies facilitate joint planning and budgeting between refugee and host services and have attracted targeted investments, including better-equipped facilities in refugee settlements [18]. However, effective integration also depends on broader health systems performance such as financing, human resources, supply chain reliability, and service readiness; which varies widely across districts and can constrain delivery of essential care. Since refugee and host communities depend on the same health services, district-level health system capacity directly shapes the quality and coverage of maternal and child health (MCH) services for both populations (Appendix: Fig. 5: Conceptual Framework). Consequently, changes in refugee service delivery can influence overall maternal and child health (MCH) indicators at the district level [10].

The Uganda Demographic Health surveys (UDHS) report of 2022 was the first to provide refugee-population estimates however, these were refugee-settlement specific hence could not guide district-level planning [19]. Comparing refugee settlements versus national targets for utilization of key MCH services antenatal care (ANC4) coverage stood at 76% which was higher than national coverage (68%), child mortality was 17 vs. 22 per 1000 live births nationally, while vaccine coverage for Measles1 was 90% vs. 83% and no vaccination was 5% vs. 2% [19]. Such discrepancies suggest that refugee health service delivery can skew district-level MCH indicators coverage which could be misleading in district planning. Therefore, granular analysis based on district estimates are essential to close this gap.

Since the integration of the health information system for Ministry of Health (MoH) with that of UNHCR in 2018 [16], health facilities in refuge settlements was synched to be reported at the respective districts. This integration allows for routine monitoring of MCH indicators using health facility data which was found a relevant alternative to frequent surveys [20, 21] and a key tool for tracking progress towards health-related sustainable development goals (SDGs) [22]. This work addresses this gap by generating service coverage estimates using routine health data during inter-survey periods, enabling more frequent monitoring at local levels and offering a reliable alternative in the absence of survey data.

In this study, we used routine health facility data to examine the differences in MCH coverage and trends between refugee-hosting (RH) and non-refugee-hosting (Non-RH) districts from 2020 to 2023 and also explored the influence of government health financing and health systems performance on MCH services utilization.

## Methods

### Study setting and data management

This was a retrospective cross-sectional study focusing on pregnant or recently pregnant women and children aged 0–23 months eligible for vaccinations. It utilized two datasets; (1) annual district expenditure data on Primary Healthcare (PHC) obtained from the Ministry of Health under the Department of Planning, Financing and Policy and (2) routine health facility data from the Uganda District Health Information System (DHIS2). Expenditure assigned to PHC focuses on essential, community-level services including maternal/child care and immunization [23]. From DHIS2, data on maternal service utilization, child immunization and health systems performance were extracted covering the period 2020–2023. Data were aggregated by district per year; districts that had data available from 2020 to 2023 were considered hence Kampala district, Terego district (among the RH districts) and the new cities (among the non-RH districts) were excluded from the analysis. Therefore, out of 142 districts, 18 district did not data for the target period, hence 134 districts were considered for analysis, of which 11 districts were RH districts and 123 were non-RH districts. Refugee-dominant (RD) districts were defined to have a refugee population exceeding 50% of the district total population; out the 11 RH districts, 5 were categorized as RD districts. Data quality checks were performed using WHO-recommended data quality metrics, including completeness of reporting, and internal consistency [24]. Since missing facility reports can bias service coverage and trends, adjustments to account for incomplete reporting were made. Without adjustment, non-reporting facilities would be assumed to have provided zero services which is an unrealistic assumption that typically underestimates true coverage. Incompleteness occurs; at the facility level, when some facilities do not submit reports at all, and at the service level, when reporting facilities submit no data for specific services or report fewer services than expected. Therefore, using Maina and colleagues’ method [25]; an adjustment factor (k = 0.25) was applied to account for lower level of service provision in non-reporting facilities assuming they provide approximately 25% of services reported by similar reporting facilities. This approach corrects for both dimensions of incompleteness and yields more reliable district-level coverage estimates [26].

### Assessment of coverage estimates

Service coverage was defined as the number of individuals who actually received the service (numerator) and the total population who needed the service (denominator).

Numerator variables included maternal health indicators: the number of pregnant women who had made an ANC visit in the first trimester, pregnant women with at least four ANC visits, number of pregnant women who delivered from health facilities, women who had Postnatal Care within 48 h and child vaccination indicators; child that had a single dose of measles vaccination, and number of children that had received third dose of Pentavalent vaccination. Health system performance indicators included district count of number health facilities, number of midwives and nurse, number of beds per facility, and number of In-patient Admissions for children under 5 years.

Denominators were calculated using census-based and facility-based methods [27]. The facility-based approach was selected because it performed best in the comparison with national estimates from the UDHS estimates. Therefore, facility-based ANC1 was used as the basis a denominator for maternal health indicators while DPT1 for the child immunization indicators.

Coverage estimates were the outcome variables including coverage of ANC visit in the first trimester (ANC1st Trimester), at-least four ANC visits (ANC4), Institutional deliveries, Postnatal Care within 48 h (PNC48hrs), single dose of measles vaccination (Measles1), and third dose of Pentavalent vaccination (DPT3).

Following the proposition by a global initiative (Countdown to 2030) aimed at tracking country-level progress in intervention coverage, the composite coverage index (CCI) a single measure was developed to provide a general picture of the coverage [28]. CCI was computed to reflect the overall performance of MCH service delivery per district [29, 30].

$$\begin{aligned}&CCI=\\ &\frac{1}{2}\left(\raisebox{1ex}{$ \left(ANC\ 1st\ Trimester\ +\ ANC4+Institutional\ Deliveries+PNC48hrs\right)$}\!\left/\!\raisebox{-1ex}{$4$}\right. \right. \\ & \quad \left.+\raisebox{1ex}{$\left(DPT3+Measles1\right)$}\!\left/\!\raisebox{-1ex}{$2$}\right.\right)\end{aligned}$$

Independent variables included the per capita expenditure, health system performance indicators such as health facilities per 1,000 population, nurses and midwives per 1,000 population, physicians per 1,000 population, bed density per 1,000 population, In-patient admissions (under-5 and total) per 1,000 population.

To enable composite performance assessments of health systems, a composite performance index was constructed from the indicators using the z-score standardization approach [31]. Higher z-scores indicated better overall district health system performance.

Per capita expenditure per district was computed as a quotient of the budget expenditure and total population size [32, 33].

### Trends analysis

MCH coverage estimates were summarized using means, and standard deviations. Trends over time (2020–2023) were visualized using line plots stratified by refugee-hosting (RH) and non-RH districts. We applied independent Student’s t-tests for each year and each MCH indicator to determine whether there were significant statistical differences by refugee-host status.

Based on the CCI, we used choropleth maps to visualize spatial-temporal trends comparing RH versus non-RH districts and refugee-dominant versus non-dominant districts for 2020 and 2023. Refugee-dominant districts were defined to have a refugee population exceeding 50% of the district total population.

Per capital expenditure had a non-normal distribution and was transformed using a natural log function.

### Relationship between coverage indicators, expenditure and refugee-host-status

Since data were collected from the same districts repeatedly over a four-year period (2020–2023), observations were not independent. Therefore, Mixed Effects Models (MEMs) were applied to account for the within-district correlation due to repeated measures. To assess the unadjusted associations, we applied separate MEM for each MCH indicator to understand the potential influence from government health financing and health system performance indicators. For adjusted associations, we applied separate MEM for each MCH indicator and a random intercept for district was included to capture unobserved heterogeneity across districts. Based on 20% significance, each adjusted MCH indicator model was generated while adjusting for per capita expenditure, composite health system performance (z-score) and years. For each of the indicators *i*, a separate MEM was generated using the equation below:

$$\begin{aligned}{Y}_{i}&={\beta}_{0}+{\beta}_{1}*\mathrm{log}({percapita}_{i})+{\beta}_{2}*{RH}_{i}+\\&{\beta}_{3}\mathrm{*}\left({year}_{i}\right)+{\beta}_{4}\mathrm{*}\left({Z-score}_{i}\right)+{\mu}_{i}+{\epsilon}_{it}\end{aligned}$$

Where; ${Y}_{i}=$ MCH indicators - CCI, ANC 1 st Trimester, ANC4, institutional deliveries, PNC48hrs,, DPT3 and Measles1.

$\mathrm{log}({percapita}_{i})=$ Log of the per capita expenditure

${RH}_{i}=$ Refugee-Host status (RH vs. Non-RH districts)

$${\beta}_{0},=$$ Overall intercept

${\beta}_{1}=$Effect of log-per capita expenditure on the coverage indicator

${\beta}_{2}=$Effect of refugee-hosting status

${\beta}_{3}=$ Effect of year

${\beta}_{4}=$Effect of composite z-score for health system performance on coverage indicators

$${\mu}_{i}=$$Random effect

Model diagnostics were conducted, including testing for Residual plots and Q-Q plots (Appendix Fig. 6). All analyses were conducted using R (v4.4.5) and Quantum GIS.

**Fig. 1:**
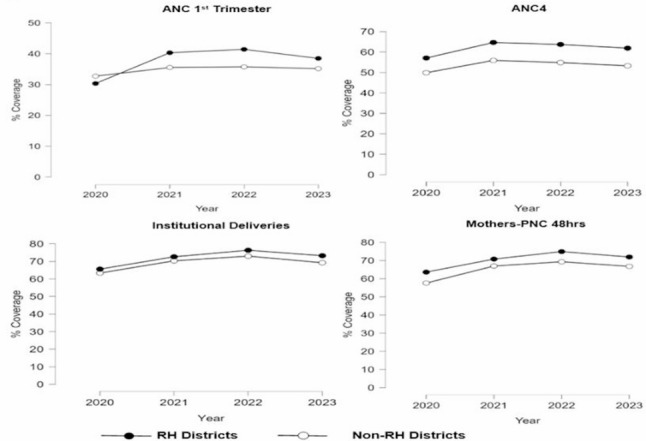
Maternal health trends (2020-2023)

## Results

Between 2020 and 2023, coverage trends in maternal health indicators were consistently higher across refugee-hosting (RH) districts than non-refugee-hosting (non-RH) districts (Fig. 1). Based on Table 4a (appendix), ANC4 coverage was statistical higher in RH compared to non-RH districts across all the years (*p* < 0.001). ANC 1 st trimester coverage gap between RH and non-RH was highest in 2022 (5.7%), while for mother’s PNC within 48 h was in 2022 (5.6%) and Institutional deliveries in 2023 (4.1%).

**Fig. 2:**
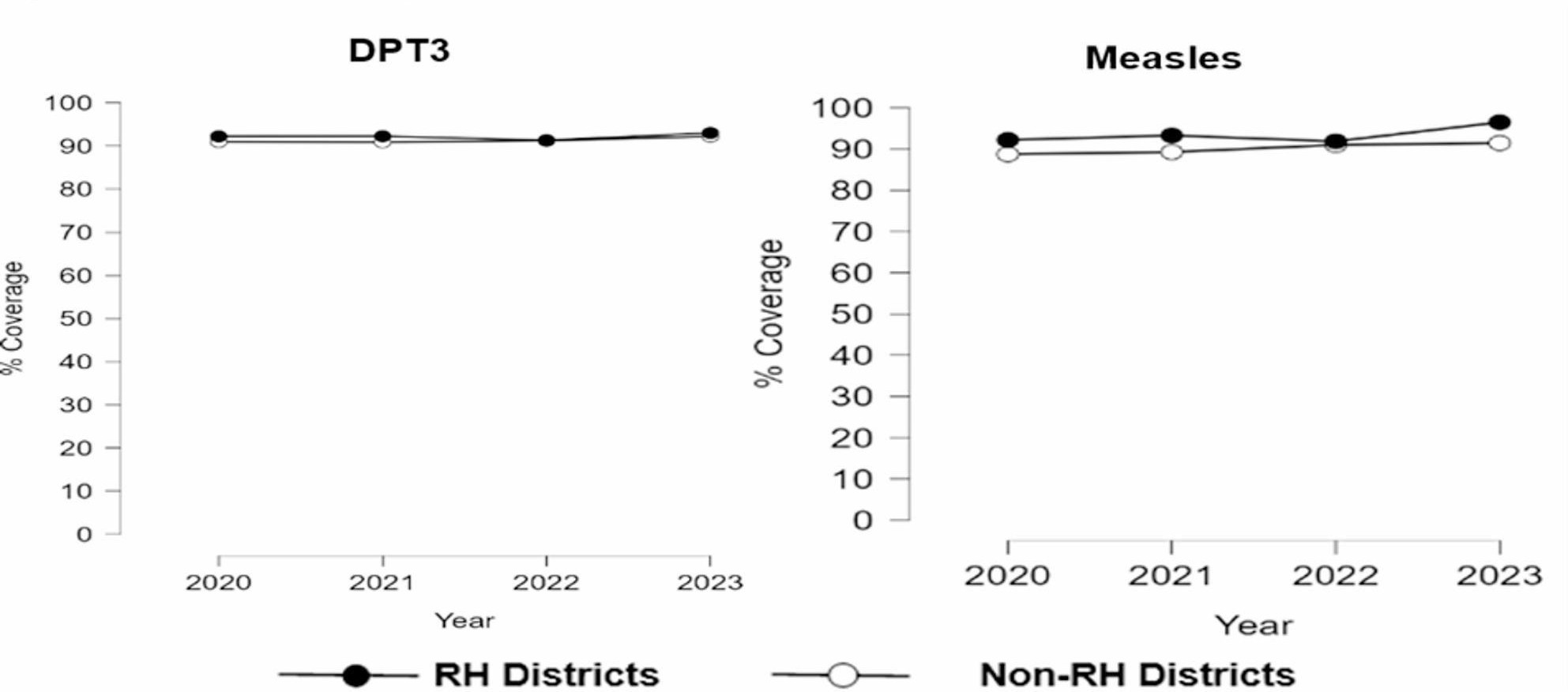
Child health trends (2020-2023)

Across the child health indicators, coverage trends were very close, however, with thin coverage gap in favour of RH districts (Fig. 2). Despite a thin coverage gap, Measles vaccination coverage was significantly higher RH districts for all years (*p* = 0.02) and coverage gap was highest in 2023 (5.0%). Trends of DPT3 coverage were slightly higher in RH in 2021 (1.4%) and 2020 (1.5%) as demonstrated in Table 4a (appendix).

**Fig. 3:**
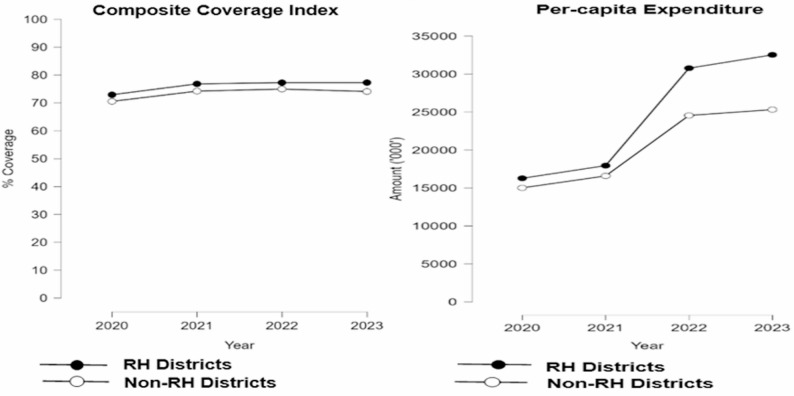
CCI and Per-capita Expenditure trends (2020-2023)

Furthermore, the per capita expenditure was consistently higher and increasing in RH districts than in non-RH districts. From 2020 to 21 and 2022-23, there was a substantial surge in expenditure, nearly doubling in both RH and non-RH districts (Fig. 3). In Table 4a (appendix), average annual expenditure was higher in RH districts by about USD 1.7 Million and USD 2.0 Million in 2022 and 2023 respectively.The coverage composite index (CCI) was slightly higher among RH districts compared to the non-RH districts (Fig. 3). The difference was statistically significant (*p* = 0.01) for all the years; RH districts had higher coverage in 2023 (3.2%), 2022 (2.3%), 2021 (2.6%) and 2020 (2.4%) as demonstrated in Table 4a (appendix).

Table 5b (in the appendix) show some summary statistics (mean and standard deviation) of MCH indicators compared over the years by Refugee-Dominant (RD) status. despite the MCH indicator coverage being higher in RD districts compared to non-RD districts, the different was not statistically significant across the individual years. However, overall financial investment in RD districts was higher than in non-RD districts (*p* = 0.007).

**Fig. 4:**
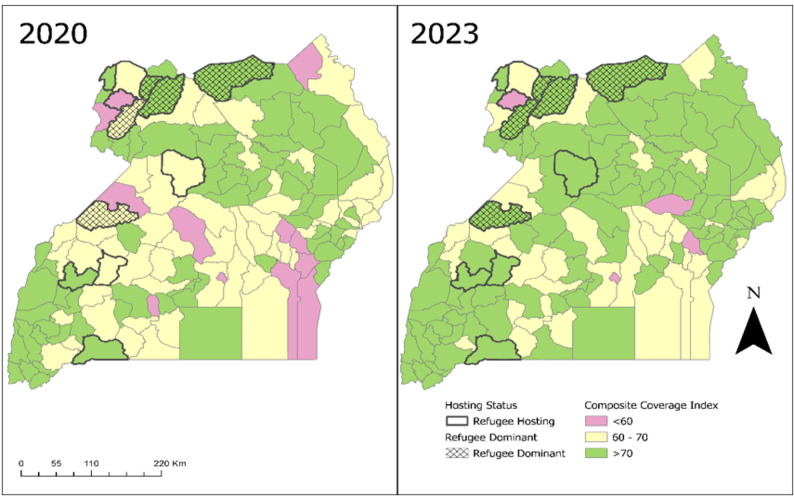
CCI saptial-temporal trends (2020-2023) comparing RH vs non-RH and RD vs non-RD districts

**Fig. 5:**
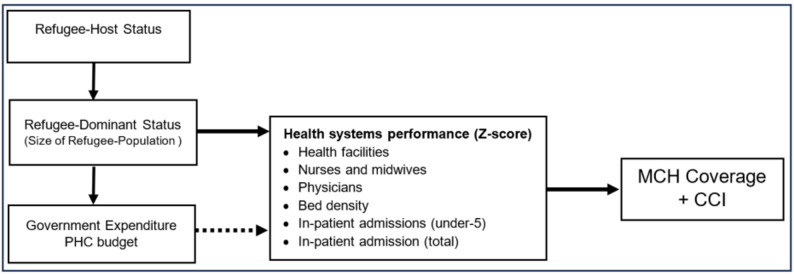
Conceptual framework

Shows the spatial-temporal trends of CCI from 2020–2023, analysis showed that coverage in the RH districts was almost as good as that in non-RH districts. 54% (6/11) RH districts had CCI above 70% while, the rest had CCI ranging between 60–70% in 2020. In 2023, there was a general improvement in the CCI; 9/11(81.8%) RH districts, had CCI above 70% (Fig. 4). Among the RH districts, 5/11 (45%) were refugee dominated (refugee population exceeded 50% of total population in the district). In 2023, CCI in all refugee dominated districts was > 70% compared to 2020 where 3/5 had > 70% coverage

Table 1 shows results unadjusted associations of several key factors with MCH coverage indicators that were statistically significant. ANC4 coverage was 8.37% point higher in RH than non-RH districts (β = 8.37; *p* = 0.016). Notably, per capita expenditure was consistently and strongly associated with higher coverage across three MCH outcomes: ANC4 (β = 5.60; *p* < 0.001), measles (β = 2.38; *p* = 0.002), and CCI (β = 4.80; *p* < 0.001). Similarly, health system performance (z-score) was significantly associated with both ANC4 (β = 4.22; *p* = 0.003) and CCI (β = 2.98; *p* < 0.001), though not with measles coverage.

Table 2 further shows the associations of the individual health systems performance indicators with MCH service coverage. The ratio of health facilities per 1,000 population was significantly associated with improved ANC4 (β = 19.87; *p* = 0.007) and CCI (β = 11.86; *p* = 0.006),. Additionally, in-patient admissions per 1,000 population (IPD) and IPD among children under five (IPD_U5) showed strong positive associations with both ANC4 and CCI. IPD was associated with an increase of 14.48% points in ANC4 (β = 14.48; *p* < 0.001) and 8.61% points in CCI (β = 8.6; *p* < 0.001). Other variables such as physician density, nurse density, and bed density were not significantly associated in the unadjusted models. Similarly RD-status did not influence coverage within the refugee-hosting districts.

**Table 1:** Unadjusted association of MCH coverage

	ANC4		MEASLES1		CCI	
	Coef (95% CI)	*p*-value	Coef (95% CI)	*p*-value	Coef (95% CI)	*p*-value
Refugee-host-status						
Non-RH	REF		REF		REF	
RH	8.37 (1.549, 15.182)	0.016	3.35 (−1.495, 8.194)	0.18	3.48 (−0.637, 7.592)	0.098
Refugee-Dominant-status						
Non-Dominant	REF		REF		REF	
Dominant	5.36 (−3.88, 14.61)	0.46	2.37 (−1.385, 6.138)	0.42	2.68 (−2.070, 7.422)	0.47
Per Capita Expenditure (Log transformation)	5.60 (3.832, 7.370)	< 0.001	2.38 (0.885, 3.880)	0.002	4.80 (3.864, 5.743)	< 0.001
z-score (Health system performance)	4.22 (1.465, 6.983)	0.003	−0.09 (−2.100, 1.904)	0.93	2.98 (1.360, 4.609)	< 0.001
Health facilities/1000	19.87 (5.559, 34.179)	0.007	−1.65 (−11.943, 8.649)	0.75	11.86 (3.322, 20.405)	0.006
Health staff/1000	0.67 (−1.531, 2.876)	0.55	−0.35 (−1.889, 1.197)	0.66	1.03 (−0.273, 2.333)	0.121
Physicians/1000	3.93 (−7.433, 15.29)	0.5	−2.93 (−10.890, 5.022)	0.47	4.57 (−2.183, 11.315)	0.18
Nurse/1000	0.90 (−2.040, 3.847)	0.55	−0.39 (−2.454, 1.673)	0.71	1.47 (−0.276, 3.208)	0.1
Bed density	15.41 (−3.610, 34.434)	0.11	0.78 (−12.665, 14.234)	0.91	9.50 (−1.849, 20.845)	0.1
IPD	14.48 (7.830, 21.120)	< 0.001	0.94 (−4.019, 5.906)	0.71	8.61 (4.644, 12.580)	< 0.001
IPD_U5	4.29 (2.031, 6.549)	< 0.001	0.79 (−0.868, 2.447)	0.35	2.46 (1.102, 3.810)	< 0.001

Table 6 presents the results of the adjusted regression analysis examining the association between MCH indicators; ANC4, Measles, CCI and key predictors; refugee-hosting status, per capita expenditure, health performance z-score index and calendar years. ANC4 coverage was 7.4% point higher in RH compared to non-RH districts (β = 7.42; 95% CI: 0.753 to 14.090; *p* = 0.029), though no significant effect was observed for Measles1 or CCI while adjusting for per capita expenditure, health performance and years. A one-unit increase in the health system performance z-score was significantly associated with a 3.6% point increase in ANC4 (β = 3.6; 95% CI: 0.729 to 6.462; *p* = 0.014) and a 2.31% point increase in CCI (β = 2.3; 95% CI: 0.642 to 3.975; *p* = 0.007), but was not significantly associated with Measles coverage while adjusting for refugee-host status, per capita expenditure, and calendar years.

Per capita expenditure was positively and significantly associated with CCI; every additional shilling spent was linked with 1.55% point increase in CCI (β = 1.55, 95% CI: 0.310 to 2.788, *p* = 0.014), but no significant effect on ANC4 or Measles1 while adjusting for refugee-host status, health performance and calendar years. Yearly comparisons, using 2020 as reference, showed significant improvements in ANC4 and CCI across all subsequent years. CCI increased significantly in subsequent years: by 2.68% points in 2021 (β = 2.68; 95% CI: 1.806 to 3.545; *p* < 0.001), 3.78% points in 2022 (β = 3.78; 95% CI: 2.965 to 4.594; *p* < 0.001), and 2.94% points in 2023 (β = 2.94; 95% CI: 2.013 to 3.858; *p* < 0.001). As well ANC4 coverage increased by 5.72% points in 2021, 4.75 in 2022, and 2.99 in 2023 (all *p* < 0.01). Between 2020 and 2023, increase in coverage of MCH indicators was significantly associated with per capital expenditure and health systems performance. RH status was significantly influenced the increase in ANC4.

**Table 2:** Effect of Government Financing, RH-status, health performance on MCH indicators

	ANC4		Measles1		CCI	
	Coef (95% CI)	*p*-value	Coef (95% CI)	*p*-value	Coef (95% CI)	*p*-value
Refugee-Hosting-status						
Non-Refugee-Hosting	REF		REF		REF	
Refugee-Hosting	7.42 (0.753, 14.090)	0.029	3.19 (−1.711, 8.102)	0.2	2.66 (−1.254, 6.575)	0.18
Per Capita Expenditure (log transformation)	1.01 (−1.361, 3.378)	0.404	1.22 (−0.828, 3.276)	0.24	1.55 (0.310, 2.788)	0.014
z-score (Health systems performance)	3.60 (0.729, 6.462)	0.014	−0.66 (−2.810, 1.483)	0.56	2.31 (0.642, 3.975)	0.007
Year						
2020	REF					
2021	5.72 (4.023, 7.419)	< 0.001	0.05 (−1.493, 1.585)	0.95	2.68 (1.806, 3.545)	< 0.001
2022	4.75 (3.155, 6.343)	< 0.001	1.63 (0.172, 3.079)	0.03	3.78 (2.965, 4.594)	< 0.001
2023	2.99 (1.193, 4.788)	0.001	2.25 (0.628, 3.870)	0.007	2.94 (2.013, 3.858)	< 0.001

## Discussion

To our knowledge, this is a premier study to systematically examine the effect of large refugee populations on host district-level MCH coverage using routine health facility data over multiple years. Prior studies (mainly cross-sectional surveys) have compared MCH service utilization among refugee versus host populations and have reported better outcomes among refugees due to targeted support from development partners [34, 35]. In Uganda, refugee-specific MCH service utilization estimates were introduced in the Uganda Demographic and Health Survey (UDHS) of 2021 [19], which focused on refugee settlements but did not provide district-level insights. Thus, there has been little empirical research exploring MCH coverage in refugee-hosting districts during inter-survey periods. Therefore, this study filled these gaps by leveraging routine DHIS2 data and government PHC expenditure records to compare MCH coverage trends between RH and non-RH districts from 2020 to 2023 while also evaluating the role of government health financing and system performance in shaping service delivery. This approach further enabled the generation of coverage estimates during periods before and between the national surveys.

This study found modest but consistent differences in MCH service coverage favoring RH districts. RH districts consistently demonstrated higher coverage for ANC 1 st trimester, ANC4, Institutional deliveries, mothers’ PNC within 48 h, and measles vaccination. Such patterns could be associated to the fact that RH districts are often targeted for additional government and donor investment under frameworks such as Uganda’s Comprehensive Refugee Response Framework (CRRF) which could be associated better infrastructure, trained health workers, and partner-supported outreach programs [36]. Such systemic enhancements boost overall district-level service availability and quality improved health outcomes [37, 38]. Similarly, districts that had dominant refugee population (refugees comprising more than 50% of district population) had higher coverage estimates. This further expounds that large refugee population may attract more funding which may be associated to better services in those districts. The overall performance indicator - composite coverage index (CCI) was higher in RH districts than non-RH districts which further reinforces the trends observed individual indicators. However, these findings were contrary to a nation-wide study in Kenya, where refugee-hosting counties: Garissa, Mandera, and Wajir had lower CCI [39]. Therefore, the need to further study and compare the construction of the CCI for better comparability.

We observed higher and increasing per capita government primary healthcare (PHC) expenditure in RH districts. Based on the regression models, PHC financingwas significantly associated with services including mothers PNC within 48 h, institutional deliveries, and ANC4. This is because improvement in PHC includes better equipped facilities, more staffing to do community outreaches. These results are consistent with prior study in Sub-Saharan Africa, which found positive correlation between MCH coverage and healthcare worker density [40].

Although refugee-hosting status was positively associated with ANC4 coverage, its effect was insignificant on the other indicators in the adjusted models. The overall MCH performance (CCI) at district-level was influenced by the government finance and health systems which indicated that PHC financing influenced coverage primarily through system strengthening. Importantly, refugee-dominance did not independently predict MCH coverage in adjusted models, hence population size alone was not a sufficient driver of service performance. Previous studies pointed to similar evidence that vertical programming in humanitarian settings could have spillover effects, improving service access for host populations as well [41, 42]. However, there was no evidence of similar benefits in child immunization outcomes, such as measles coverage, pointing to possible gaps in program reach or differences in delivery mechanisms for maternal versus child health services.

When compared to data from the recent UDHS 2022 report, our findings in RH districts indicated lower coverage for ANC4 (53% vs. 76%) and institutional deliveries (69% vs. 96%), but, almost similar for measles vaccination (91% vs. 90%) [19]. These discrepancies could be attributed to the difference in the geographical scope of data collection: the survey focused only on refugee settlements while our study analysed coverage at district level. This distinction is important under Uganda’s integrated model, where service improvements in refugee areas may also influence access for host populations.

Financial investment at the district level emerged as a key enabler of improved coverage. Although its influence was strongest in unadjusted models, government health expenditure remained positively associated with composite service coverage even after accounting for refugee-hosting status, year, and health system characteristics. This supports earlier findings from low- and middle-income countries (LMICs), which suggest that increased per capita health spending was associated with better service coverage, particularly when combined with system strengthening efforts [43, 44]. Importantly, our analysis reinforced the role of health system capacity which was measured through a standardized index (z-score) combining infrastructure, workforce, and utilization as a significant determinant of both maternal care and overall service performance. Using z-score approach to capture system readiness was consistent with methods employed in health system performance evaluations in the U.S. and LMICs, allowing for meaningful comparisons across diverse service domains [45].

Therefore, such investments have positively impacted health outcomes for both refugees and host communities, particularly in the West Nile region [36, 46]​. ​Addressing these perceptions was critical to fostering trust and inclusivity in health service delivery. Generally, this study findings highlight the value of Uganda’s integrated policy approach, which allows for joint service planning, equitable access, and efficient resource use. They also demonstrated the utility of routine health facility data in identifying disparities and tracking progress in settings where refugee and host populations were served through the same health system.

### Strengths

Among the strengthens of this study was its novelty, it was the first study to systematically examine the effect of large refugee populations on host district MCH coverage using routine health facility data over multiple years. Additionally, routine health facility data demonstrated improved data quality in terms of completeness and comprehensiveness over time, ensuring reliability for the analysis. The comparative nature of analysis that examines RH vs. Non-RH districts and RD vs. non-RD districts highlights geographic disparities and informs targeted interventions, thus making the study findings relevant in the current settings. In addition, the study used mixed effects modelling to cater for repeated measures across districts gave the study more statistical rigour. The study integrated financial data; government health budget and per capita expenditure per district provided a clearer understanding of financial determinants of health service coverage. Finally, the study entailed the use of Composite Indices (CCI and health system performance z-score) which enabled comprehensive assessment of service delivery and system readiness.

### Limitations

Our analysis had limitations. First, it excluded districts that had attained city status after 2020, primarily due to structural and administrative changes which introduced inconsistencies in routine health data reporting. Such an exclusion limits the generalizability of our findings to urban settings, yet these present distinct health service delivery dynamics, such as higher population mobility, different health-seeking behaviors, and more complex health system structures. Additionally, factors such as population growth and internal migration patterns were not included, yet they could influence service demand and health system strain thus potentially confounding district-level comparisons. Second, the financial data used in the analysis focused on reported government health expenditure on Primary Healthcare (PHC) per district, which did not fully capture the breadth of resources flowing into refugee-hosting districtsas it did not reflect off-budget support from local or international donors. Refugee-hosting districts are known to receive substantial supplementary support, including additional human resources, health infrastructure investment, and programmatic oversight. These contributions likely improve district-level health system performance but were not quantifiable in our analysis, thereby limiting our ability to directly attribute improvements in MCH service coverage to formal government spending alone.

Third, our district-level approach did not disaggregate data between refugee and host populations, limiting our ability to assess within-district equity. As such, higher MCH coverage observed in refugee-hosting districts could reflect improvements among refugees, host communities, or both. Moreover, important structural and contextual variables such as socio-economic status (SES), urbanization and education levels were not incorporated in the models which confounders may influence service coverage.

## Conclusion

RH districts in Uganda demonstrated modestly higher MCH coverage compared to non-RH districts from 2020 to 2023. Despite the magnitude of differences being small, this trend was notably consistent across multiple MCH indicators and years.

These findings suggested that targeted investments in RH districts, aimed at supporting both displaced and host populations could contribute to broader improvements in health service delivery. However, due to the absence of sub-population data, we were unable to determine whether these gains are equitably distributed between refugee and host communities. Therefore, future efforts should prioritize utilization of disaggregated data to better understand population-specific impacts and ensure balanced funding approaches to promote equitable health outcomes across all districts.

### Program and policy implications

The combination of the modestly higher MCH service coverage in RH districts and consistently higher health expenditures emphasizes the potential role of targeted health financing in strengthening service delivery, even in the contexts of displaced populations. Therefore, while efforts to improve health service delivery for refugees should continue, it is equally important to monitor and address coverage disparities to maintain equity across all districts.

While the inability to distinguish refugee from host populations limits the conclusions we can draw about equity or subgroup outcomes, the results nonetheless highlight the importance of sustained and equitable financing for district health systems. Future investments should aim to improve data systems to allow for sub-population disaggregation and incorporate contextual factors such as Socio-Economic-Status and health system capacity. Such improvements will enable more precise monitoring thus facilitating both refugee and host populations to benefit equitably from health interventions.

### Recommendations

The government of Uganda under the Ministry of health should explore mechanisms to ensure equitable resource distribution while addressing the specific needs of RH districts. For improved universal government coverage in Uganda, interventions should aim to enhance coverage for maternal and child health services in Non-RH districts, especially those with lower per capita expenditures.

Further research ought to investigate the underlying reasons for the negative association between budget expenditure and maternal health indicator-ANC1 coverage and explore other unmeasured determinants of coverage disparities. Furthermore, continued emphasis on improving routine health data quality will enhance the accuracy of future analyses and policy decisions.

Since UDHS report estimates were conducted within settlements, future research should compare these estimates with on health facility data per settlement to provide a more comprehensive picture of coverage. Moreover, continuous monitoring of coverage and equity across all districts, including both RH and non-RH areas, is essential to ensure that the health system remains fair and responsive to the needs of all populations in Uganda.

## Figures and Tables

**Fig. 6:**
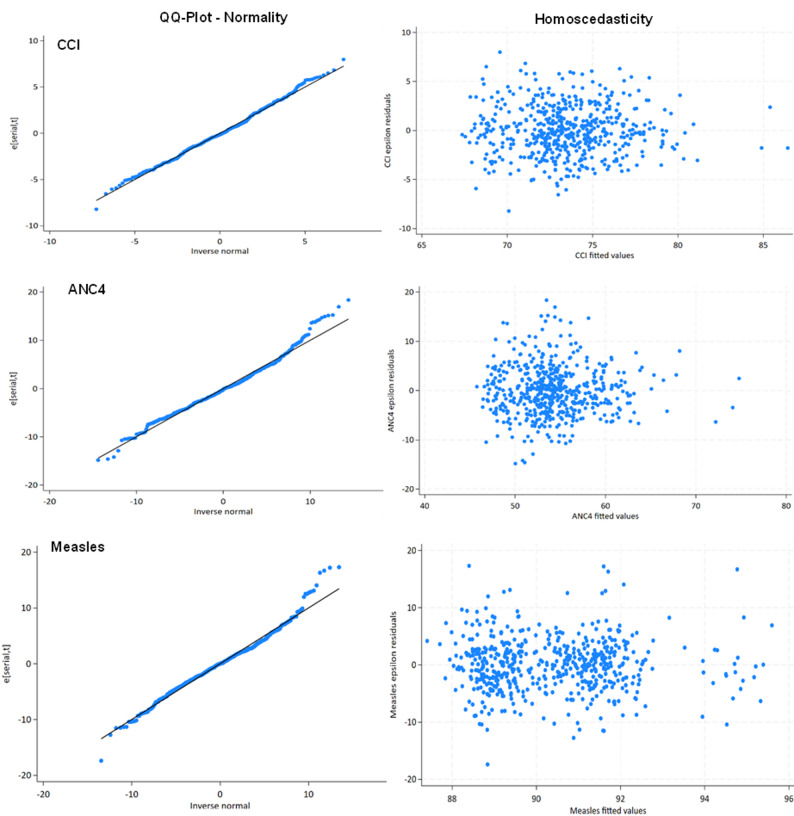
Model diagonstics

## Data Availability

The dataset are property of Government of Uganda and are available upon request from the Ministry of Health.

## Appendix

### Derivation of denominators

The census-based approach derived the total live births from the projected total population and the crude birth rate (CBR) from the latest Demographic Health Survey (UDHS 2022). The expected total number of births, deliveries, and pregnssancies was estimated by applying the stillbirth rate, the proportion of multiple births (twins, triplets) and the proportion of pregnancies ending in early fetal death, while the expected number of infants was estimated using an expected number of live births and the neonatal mortality rate to calculate the service-based denominators. Similar adjustments to the census-based approach were applied to estimate the expected number of total pregnancies, deliveries, births, and infants.

Using the census- and health service-based denominators, we produced four coverage estimates for institutional delivery and DPT3. The best denominators that produced national estimates were assessed based on the least absolute difference from the UDHS estimates. Therefore, facility-based denominators ANC1 (having atleast ANC visit throughout the pregnancy period) and were institution delivery coverage indicators (ANC4, Institutional deliveries and PNC48) and DPT3, respectively.

Overall, the data quality score showed improvement in the routine health facility data since 2020 with a slight decrease in 2021. Specifically, completeness as a data quality consistently increased over the year thus indicating a positive performance trend and good comprehensive reporting. (Appendix: Table 3).

**Table 3:** Data Quality Scores

Data quality metrics	2020	2021	2022	2023
**Completeness of monthly facility reporting**				
% of expected monthly facility reports (national)	95	96	97	97
% of districts with completeness of facility reporting > = 90%	87	88	90	92
**Annual data quality score**	89.3	89.1	87.7	87.8

**Table 4:** a: Descriptive Statistics comparing MCH coverage across refugee-host status over the years

Year	2020		2021		2022		2023		Overall
RH status	Non-RH	RH	Non-RH	RH	Non-RH	RH	Non-RH	RH	
N	123	11	123	11	123	11	123	11	p-value
	**Mean (± SD)**	**Mean (± SD)**	**Mean (± SD)**	**Mean (± SD)**	**Mean (± SD)**	**Mean (± SD)**	**Mean (± SD)**	**Mean (± SD)**	
*ANC 1 st Trimester*	32.7(10.8)	30.3(8.2)	35.5(10.6)	40.3(12.4)	35.7(10.0)	41.4(12.7)	35.2 (9.6)	38.5(11.8)	0.082
*ANC4* ^*1,2,3*^	49.9(11.7)	57.1(12.3)	55.9(12.9)	64.7 (12.9)	54.9(12.0)	63.7(12.5)	53.3(11,6)	61.9(12.2)	< 0.001
*Institutional Deliveries*	63.4(16.0)	65.6(9.8)	70.3(17.5)	72.6(11.7)	73.0 (17.9)	76.3(11.9)	69.2(16.9)	73.3(11.0)	0.265
*Mother’s PNC48hrs*	57.6(15.9)	63.6(10.5)	67.0 (17.4)	70.8(11.5)	69.3(17.4)	74.9(11.7)	66.8(16.8)	71.9(10.1)	0.055
*DPT 3*	91.0 (5.0)	92.2(5.6)	90.9(4.7)	92.3(3.2)	91.2(5.3)	91.3(3.2)	92.2(4.3)	93.0(3.5)	0.23
*Measles**	88.7(9.4)	92.2 (5.6)	89.3(9.5)	93.3(5.7)	91.0(9.2)	91.9(6.3)	91.5(8.9)	96.5(9.5)	0.02
*CCI**	70.4 (7.2)	73.2 (6.2)	73.6 (7.4)	77.4 (6.9)	74.7 (7.1)	77.9 (6.1)	74.0 (7.1)	78.1 (6.3)	0.01
*Per capita Expenditure* ^*2,3**^	1357.7 (642.7)	1377.2 (739.6)	2092.6 (1038.4)	2778.0 (1976.4)	1962.3 (1052.6)	3111.4 (2474.3)	2254.0 (1135.2)	3102.4 (2155.7)	< 0.001

^1,2,3,^* statistically significant at < 0.05 for the year 2021, 2022, 2023 and across all years respectively

**Table 5:** b: Descriptive Statistics comparing MCH coverage across refugee-dominant status over the years

Year	2020		2021		2022		2023		Overall
Refugee-Dominant status	Non-RD	RD	Non-RD	RD	Non-RD	RD	Non-RD	RD	
N	6	5	6	5	6	5	6	5	p-value
	**Mean (± SD)**	**Mean (± SD)**	**Mean (± SD)**	**Mean (± SD)**	**Mean (± SD)**	**Mean (± SD)**	**Mean (± SD)**	**Mean (± SD)**	
*ANC 1 st Trimester*	27.1 (6.02)	34.3 (9.45)	38.1 (8.01)	43.0 (17.05)	38.6 (7.62)	44.8 (17.56)	33.9 (4.90)	44.0 (15.82)	0.048
*ANC4*	54.3 (10.23)	60.5 (14.85)	62.8 (10.53)	67 (16.34)	61.8 (13.35)	66 (12.59)	58.8 (13.05)	65.6 (11.16)	0.18
*Institutional Deliveries*	63.8 (8.50)	67.8 (11.86)	73.0 (12.13)	72.2 (12.49)	76.9 (14.61)	75.7 (9.40)	73.2 (13.45)	73.4 (8.80)	0.88
*Mother’s PNC48hrs*	60.5 (9.30)	67.4 (11.62)	70.5 (11.46)	71.1 (12.83)	74.5 (14.48)	75.5 (8.98)	71.1 (12.05)	72.9 (8.29)	0.46
*DPT 3*	91.9 (2.13)	92.6 (2.52)	91.8 (2.24)	92.7 (4.38)	90.8 (3.45)	92 (3.07)	93.2 (1.94)	92.7 (5.12)	0.57
*Measles*	90.8 (3.09)	93.9 (7.73)	93.4 (4.28)	93.1 (7.66)	89.1 (6.04)	95.2 (88.8)	96.3 (7.41)	96.8 (12.46)	0.27
*CCI*	71.4 (4.56)	75.4 (7.70)	76.9 (5.31)	78.1 (9.12)	76.4 (5.79)	79.6 (6.64)	77.0 (6.34)	79.3 (6.73)	0.19
*Per capita Expenditure*	1116.4 (394.59)	1690.2 (973.80)	1892.4 (776.54)	3840.8 (2534.39)	2169.8 (478.72)	4241.4 (3477.49)	2285.2 (503.17)	4083.1 (3016.19)	0.007

**Table 6:** Unadjusted association with MCH performance

	ANC1		INST		PNC48		DPT3	
	Coef (95% CI)	*p*-value	Coef (95% CI)	*p*-value	Coef (95% CI)	*p*-value	Coef (95% CI)	*p*-value
Refugee-host-status								
Non-RH								
RH	2.85 (−2.911, 8.619)	0.33	2.98 (−6.818, 12.776)	0.55	5.16 (−4.440, 14.755)	0.292	0.88 (−1.566, 3.329)	0.48
Per Capita Expenditure	3.94 (2.409, 5.476)	< 0.001	9.52 (7.49, 11.550	< 0.001	12.65 (10.485, 14.809)	< 0.001	0.45 (−0.361, 1.254)	0.28
z-score	1.89 (−0.460, 4.233)	0.12	9.04 (5.33, 12.75)	< 0.001	934 (5.726, 12.947)	< 0.001	−0.21 (−1.217, 0.790)	0.68
Health facilities/1000	12.8 (0.797, 24.835)	0.04	33.79 (13.890, 53.682)	0.001	31.65 (12.032, 51.273)	0.002	0.04 (−5.139, 5.217)	0.99
Health staff/1000	0.24 (−1.586, 2.074)	0.79	4.19 (1.169, 7.210)	0.007	4.13 (1.166, 7.100)	0.006	−0.15 (−0.927, 0.624)	0.7
Physicians/1000	2.53 (−6.914, 11.976)	0.6	18.81 (3.105, 34.520)	0.019	18.24 (2.805, 33.684)	0.02	−0.56 (−4.566, 3.447)	0.78
Nurse/1000	0.40 (−2.053, 2.843)	0.75	5.87 (1.84, 9.897)	0.004	5.78 (1.819, 9.737)	0.004	−0.22 (−1.255, 0.820)	0.68
Bed density	7.70 (−8.200, 23.60)	0.34	21.17 (−5.640, 47.975)	0.12	28.09 (1.947, 54.224)	0.04	1.03 (−5.733, 7.785)	0.77
IPD	4.85 (−0.980, 10.681)	0.1	24.13 (15.029, 33.224)	< 0.001	25.29 (16.483, 34.104)	< 0.001	−0.87 (−3.359, 1.625)	0.49
IPD_U5	1.63 (−0.327, 3.579)	0.1	6.04 (2.862, 9.227)	< 0.001	6.55 (3.458, 9.639)	< 0.001	−0.22 (−1.057, 0.614)	0.6
